# Early Feeding Factors and Eating Behaviors among Children Aged 1–3: A Cross-Sectional Study

**DOI:** 10.3390/nu14112279

**Published:** 2022-05-29

**Authors:** Daria Masztalerz-Kozubek, Monika A. Zielinska-Pukos, Jadwiga Hamulka

**Affiliations:** Department of Human Nutrition, Institute of Human Nutrition Sciences, Warsaw University of Life Sciences (SGGW-WULS), 02-776 Warsaw, Poland; daria_masztalerz_kozubek@sggw.edu.pl (D.M.-K.); jadwiga_hamulka@sggw.edu.pl (J.H.)

**Keywords:** breastfeeding, complementary feeding, complementary feeding method, complementary foods, mealtime environment, eating behaviors, infant feeding practices, CEBQ

## Abstract

Early nutrition plays a crucial role not only in providing essential nutrients for proper child development, but may also be an important step in creating desirable eating behaviors, which can be transmitted into adulthood. The aim of this study was to assess possible links between early feeding factors, such as breastfeeding, complementary feeding (timing and method) as well as types of complementary foods and mealtime environment during the first three months of complementary feeding and eating behaviors in children aged 1–3 years old. This cross-sectional, online survey involved 467 mothers of toddlers aged 1–3 years old from the whole of Poland. The questionnaire consisted of questions about early feeding and the Children’s Eating Behavior Questionnaire (CEBQ). The adjusted linear regression model revealed that longer duration of any breastfeeding was negatively related to enjoyment of food (EF), desire to drink (DD) and positively related to satiety responsiveness (SR) and slowness in eating (SE) subscales. Moreover, offering homemade complementary foods more often than commercial may be related to higher SR. Eating meals during distraction seems to be negatively associated with EF, and positively with DD and SE subscales. Our study highlights possible links between early feeding factors and toddlers’ eating behaviors, so further investigation, also including dietary factors, is needed.

## 1. Introduction

Nutrition during early life is an important factor in shaping food preferences and eating behaviors that can be transferred into childhood and beyond [1,2]. Development of healthy eating habits influences later health and may prevent obesity and other non-communicable diseases which are considered as social and public health problems [2,3].

Among early nutrition factors, breastfeeding and complementary feeding play a major role. Exclusive breastfeeding for the first 6 months of life is a desirable goal in infants’ nutrition and should be continued as complementary foods are introduced, as long as mutually desired by mother and infant [4,5]. Breastfeeding, besides having many health benefits both for mother and child [6], may also contribute to establishing food preferences and eating behaviors [1,7,8,9].

Complementary feeding is the next stage in infant’s feeding. It is possible to start introducing solids between 17 and 26 weeks [10,11]; however, exclusive breastfeeding for 6 months is a gold standard in infants’ nutrition and, during this time, breast milk provides all the essential nutrients in the majority of children [10]. However, besides timing, psychological and neurological maturation is also crucial [10]. Complementary feeding is a gradual process of introduction foods and beverages other than breastmilk/formula and it typically continues to 24 months [12,13]. Complementary feeding, besides its role in providing nutrients, is also an important period in the acquirement of an optimal eating behavior and healthy eating habits [12]. Responsive feeding, in which the child leads the feeding interaction, in contrast to it being the parent’s responsibility over what, when and where a child is fed, seems to play an important role in the context of shaping food preferences and establishing desirable eating behaviors [13,14]. 

There is no concise recommendation about method of complementary feeding or type of complementary foods [10]. Over the last year, interest in baby-led weaning method (BLW) has grown substantially, suggesting much promise in relation to possible benefits of BLW to infant eating behaviors; however, results are inconclusive [14]. To the best of our knowledge, the number of studies focusing on types of complementary foods in relation to eating behavior is scarce. On the one hand, WHO reports suggest that the nutrition quality of commercial complementary foods may be inadequate [15,16]. On the other hand, there is a possible risk of offering unsuitable family foods, with the addition of salt/sugar.

Another factor, besides timing and method of introducing complementary foods, that may be important in creating healthy eating behaviors is mealtime environment. Previous studies have suggested that frequent family mealtimes may be associated with more desirable eating behaviors, better diet quality as well as decreased risk of overweight/obesity and eating disorders [7,17,18]. In addition, meal consumption during distraction, such as watching television, may be a risk factor for developing unhealthy food habits [19].

Recent studies have analyzed eating behaviors in the context of early feeding factors; however, results are inconclusive [8,20,21,22,23,24,25,26,27,28]. Moreover, previous studies sometimes focused on selected early feeding factors such as breastfeeding [24,28], complementary feeding [29,30] or mealtime habits [18,31,32] only.

The aim of the present study was to assess possible links between breastfeeding, complementary feeding (timing and method) as well as types of complementary foods and mealtime environment during the first three months of complementary feeding and eating behaviors in children aged 1–3 years old.

## 2. Materials and Methods

### 2.1. Study Design and Participants

The study followed the ethical standards recognized by the Declaration of Helsinki and was approved by the Ethics Committee of the Faculty of Human Nutrition and Consumer Science, Warsaw University of Life Sciences, Poland, on 19/07/2019 (Resolution No. 45/2019).

The study was designed as a cross-sectional study among mothers of children aged 1–3 years old from Poland. Data related to the study were collected in 2020–2022, with the use of the CAWI (Computer-Assisted Web Interview) method. Mothers were recruited through social media. The questionnaire was published in parenthood-specific discussion boards using the Google Forms web survey platform. The link to the online survey was shared through social media, such as Facebook, Instagram, and WhatsApp, and by personal contacts of the research group members. Participants received information about the anonymity of the study, the voluntary nature and the possibility to stop their participation at any study stage.

The inclusion criteria were formulated as follows:

- Internet access;
- Living in Poland;
- Willingness to participation in the study.

The exclusion criteria were:

- Child’s age less than 12 or more than 36 months;
- Living abroad;
- Lack or incomplete data about breastfeeding, complementary feeding, and maternal anthropometry;
- Gestational age less than 23 or more than 44 weeks.

**Figure 1:**
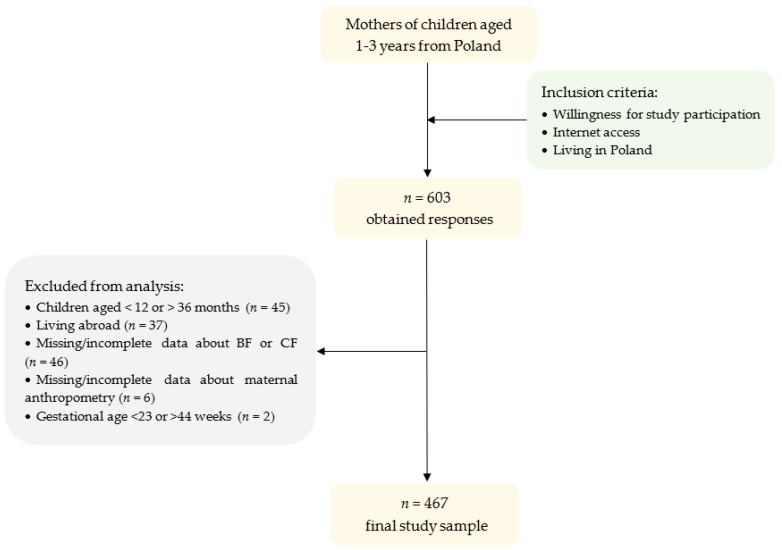
Flowchart presenting exclusion criteria and study population. BF—breastfeeding; CF—complementary feeding.

The questionnaire was completed by 603 participants and 467 (77% of initial sample) of them were included in the final analysis (Figure 1). Participants were excluded due to lack of or incomplete data, living abroad, child’s age (less than 12 or more than 36 months) and extreme gestational age.

### 2.2. Questionnaire

The questionnaire comprised questions about early and current feeding practices, birth-related and demographic data, as well as questions regarding child and maternal anthropometry. One of the parts of the survey was the Children’s Eating Behavior Questionnaire (CEBQ).

#### 2.2.1. Early Feeding Practices

Mothers were asked about milk feeding practices—whether they ever breastfed and for how long. Information about duration of any and exclusive breastfeeding was gathered. Due to the definition of exclusive breastfeeding [33], if an infant received water or any other food/drink product during the declared period of exclusive breastfeeding, the duration was adequately corrected. 

Additionally, mothers were asked about the first 3 months of complementary feeding period, such as age at when they introduced particular food/drink products. On this basis, we calculated age when infants started complementary feeding. Time of introducing complementary feeding was defined as the month when children received for the first time any other than breastmilk/formula product (including water; not applying to water that was an ingredient of formula milk). Among infants who were born prematurely, we reported data expressed in corrected age. Age at complementary feeding introduction was categorized as (1) complementary feeding started before 4 months, (2) before 4 and 6 months, (3) after 6 months. Information about method of complementary foods introduction was also gathered. Baby-led weaning (BLW) was defined as solely or mostly baby feeding themselves, mixed method as about half spoon-feeding by an adult and half baby feeding themselves, and tablespoon feeding (TSF) as mostly or solely spoon-fed by an adult. Data about types (commercial baby foods and drinks, homemade adapted for infants, family foods) of complementary foods and mealtime environment (with family, during watching TV, distraction, playtime) were also examined.

#### 2.2.2. Feeding Practices and Toddlers’ Dietary Habits in the Last 3 months

We also asked about current feeding practices, such as mentioned above mealtime environment, frequency of consumption of selected food items and use of added salt and sugar in the toddlers’ diets.

##### Children’s Eating Behavior Questionnaire

Current eating behaviors were assessed using the Children’s Eating Behavior Questionnaire (CEBQ) completed by mothers. It is a 35-item tool, where respondents rate each item on a 5-point Likert scale from 1 (never) to 5 (always). Results for each subscale were calculated as the mean from all items in the given subscale. 

CEBQ is a psychometric tool for assessing eating behaviors in children and was originally developed and validated by Wardle et al. [34]. Since then, CEBQ in original or modified versions has been used in multiple studies, involving wide age ranges of study subjects (from 12 months [35] up to 16 years old [20,36,37,38,39,40,41,42,43]). Originally, the CEBQ included eight subscales, four of them representing “food approach” traits—food responsiveness (FR), enjoyment of food (EF), emotional overeating (EOE), and desire to drink (DD), with the remaining four representing “food avoidance” eating traits—satiety responsiveness (SR), food fussiness (FF), slowness in eating (SE) and emotional undereating (EUE) [44,45]. Appetitive traits can vary with age; nonetheless, some studies showed good continuity/stability of selected eating behaviors over time [42,46,47]. In this research we used the polish version of CEBQ adapted by Czepczor-Bernat and Brytek-Matera [36].

#### 2.2.3. Children and Maternal Anthropometry

Information about current toddlers’ body weight and height were gathered. On this basis, BMI z-scores were calculated using the WHO Anthro Survey Analyser [48] and interpreted according to WHO criteria [49]. Mothers were also asked about their weight and height; based on these data, maternal BMI was calculated and interpreted based on the WHO [50].

#### 2.2.4. Birth-Related Data

In this part of the survey, mothers were asked about type of pregnancy (singleton or multiple), gestational age (in weeks) and birth parameters. Children who were born before 37 weeks of pregnancy were categorized as ‘premature’ and then corrected age was calculated. On the basis of birthweight and gestational age, we calculated birthweight to gestational age centiles, using the INTERGROWTH-21st Neonatal Size Calculator [51] and interpreted results as follows: small for gestational age (SGA) as lower than 10th percentile, appropriate to gestational age (AGA) as 10th–90th percentile and large for gestational age (LGA) as higher than 90th percentile.

#### 2.2.5. Toddlers’ Health and Development

Data about toddlers’ health conditions, such as occurrence of food allergies, hyper- and hypotonia, atopic dermatitis, sensory integration disorders, sleep duration, screen time, attendance to daycare and maternal opinion about toddlers’ body weight were obtained.

#### 2.2.6. Demographic Data

The following data were gathered: parental age and education level (further categorized as follows: (1) less than 29 years, (2) 30–34 years, (3) 35 years or more and (1) high school or lower, (2) university, respectively), place of residence—size and region of the country, further categorized according to the gross domestic product (GDP) per capita in purchasing power standards in relation to EU-27 average [52] ((1) 51–100%—Lower Silesian, Kuyavian-Pomeranian, Lublin, Lubusz, Łódź, Lesser Poland, Opole, Subcarpathian, Podlaskie, Pomeranian, Silesian, Holy Cross, Warmian-Masurian, Greater Poland, West Pomeranian voivodships; (2) 101–130%—Masovian voivodship), number of persons and children in the household. For the children, information about current age (further categorized into three age groups: (1) 12–18 months, (2) 19–24 months, (3) 25–36 months) and sex was obtained.

### 2.3. Statistical Analysis

Qualitative data were reported as a percentage (%) and numbers (*n*) and quantitative data as a mean ± standard deviation (SD). After checking the normality of distribution by Kolmogorov–Smirnov test, we used U-Mann–Whitney or Kruskal–Wallis tests to check differences between variables.

Early feeding practices, types of complementary food and mealtime environment in the first three months of complementary feeding patterns were determined using the *k-means* algorithm. Early feeding pattern included data about exclusive breastfeeding duration, age at complementary feeding introduction and current breastfeeding. Three clusters were selected: (1) longer ABF, characterized by current breastfeeding but lower exclusive breastfeeding duration (4.1 ± 2.4 months) and complementary feeding at 5.6 ± 0.9 months; (2) formula, with very low exclusive breastfeeding duration (0.3 ± 0.7 months), lack of current breastfeeding and introduction of complementary foods at 4.3 ± 1.9 months; (3) longer EBF, characterized by lack of current breastfeeding but longer duration of exclusive breastfeeding (5.4 ± 0.9 months) and complementary feeding introduction at 5.6 ± 0.9 months (Table S1). In the types of complementary food pattern, we selected two clusters: (1) homemade, characterized by more frequent consumption of homemade meals cooked especially for baby and family meals adjusted for babies, and (2) commercial, characterized by more frequent consumption of commercial foods for babies (cereals, fruit/dinner/soup jars) (Table S2). In the mealtime environment (during first three months of complementary feeding) pattern, three clusters were selected: (1) distracted, in which infants ate more often while doing other activities (such as watching TV, playtime, or were distracted by parent); (2) separated, characterized by more frequent meal consumption at different times than other family members; (3) family, characterized by more frequent meal consumption with the rest of family (Table S3).

## 3. Results

In the study population, nearly half of the mothers were 30–34 years old (47.5%), most of them had university education (85.2%) and lived in macroeconomic region with 51–100% of GDP EU-27 average (77.1%; Table 1). Most of the children were born in term (91.0%), and had appropriate to gestational age birthweight (77.1%; Table S4). One-third (31.3%) of toddlers were exclusively breastfed for at least 6 months and nearly 40% of mothers were currently breastfeeding. More than 80% of the children were introduced to complementary foods between 4 and 6 months.

### 3.1. Sociodemographic Factors and Eating Behaviors

Results regarding the Children’s Eating Behavior Questionnaire are listed in Table 1. The highest score was observed on the EF subscale (3.54 ± 0.75), whereas the lowest was observed on the EOE subscale (1.44 ± 0.51). We did not notice any differences between maternal age, macroeconomic region residence or child’s gender and CEBQ measures (Table 1). However, children of mothers with a higher education level scored higher on the FF subscale compared to children of mothers with a lower education level (2.65 ± 0.90 vs. 2.37 ± 0.83, *p* ≤ 0.05). We also observed differences between child’s age and EF, SR, SE and FF subscales. The youngest children scored higher on the EF subscale (3.73 ± 0.69) and lower on the FF subscale (2.26 ± 0.81) when compared to those aged 19–24 (3.43 ± 0.80; 2.78 ± 0.90) and 25–36 months (3.42 ± 0.73, *p* ≤ 0.001; 2.84 ± 0.87, *p* ≤ 0.001, respectively). On the SR and SE subscales, differences were observed only between children aged 12–18 and 19–24 months. Younger children scored lower on SR (2.82 ± 0.61) and SE (2.69 ± 0.59) subscales than older children (3.03 ± 0.67, *p* ≤ 0.05; 2.86 ± 0.64, *p* ≤ 0.05, respectively) (Table 1).

### 3.2. Birth-Related Factors, Maternal BMI and Eating Behaviors

Children with lower birthweight scored lower on the FR subscale (1.64 ± 0.45) than those with a higher birthweight (2.07 ± 0.75, *p* ≤ 0.05). We did not notice any other differences between pregnancy duration, birthweight to gestational age categories or maternal BMI and eating behaviors (Table S4).

### 3.3. Early Feeding Factors and Eating Behaviors

Table 2 lists the data on early feeding pattern, age and method of complementary feeding introduction, types of complementary food and mealtime environment patterns. We noticed differences between early feeding pattern and results in FR (*p* ≤ 0.01), DD (*p* ≤ 0.01) and SR (*p* ≤ 0.01) subscales and between age at complementary feeding introduction and scores in SE subscale (*p* ≤ 0.05). Method of complementary feeding introduction was a factor that varied on the EF (*p* ≤ 0.001) and FF (*p* ≤ 0.001) subscales. We also observed differences between types of complementary food pattern and results on the SR subscale (*p* ≤ 0.05), as well as mealtime environment pattern and scores on the EF (*p* ≤ 0.001), DD (*p* ≤ 0.05), SE (*p* ≤ 0.05) and FF (*p* ≤ 0.001) subscales.

### 3.4. Early Feeding Factors Associating with the CEBQ Results

Results of linear regression analysis are presented in Table 3 (multivariate model) and Table 4 (model adjusted for children age, gender and maternal education). The univariate model is included in Supplementary Materials (Table S5). 

#### 3.4.1. Early Feeding Pattern

In the univariate regression analysis, we noticed a negative association between pattern with a longer duration of any breastfeeding and scores in FR (*β* = −0.160, 95% CI: −0.260– −0.060, *p* ≤ 0.01) and DD (*β* = −0.194, 95% CI: −0.294– −0.094, *p* ≤ 0.001) subscales. Moreover, formula feeding pattern was positively associated with higher scores on the DD subscale (*β* = 0.119, 95% CI: 0.019–0.219, *p* ≤ 0.05; Table S5). 

In the multivariate model, association with FR subscale was no longer significant, whereas associations with DD and SR subscales remained significant both in multivariate (DD: longer ABF *β* = −0.187, 95% CI: −0.289– −0.085, *p* ≤ 0.001, formula *β* = 0.114, 95% CI: 0.013–0.215, *p* ≤ 0.01; SR: longer ABF *β* = 0.117, 95% CI: 0.014–0.220, *p* ≤ 0.05; Table 3) and adjusted models (DD: longer ABF *β* = −0.183, 95% CI: −0.292– −0.074, *p* ≤ 0.01, formula *β* = 0.109, 95% CI: 0.006–0.212, *p* ≤ 0.05; SR: longer ABF *β* = 0.158, 95% CI: 0.050–0.267, *p* ≤ 0.01; Table 4).

Furthermore, in the multivariate model, longer ABF pattern turned out to be associated with lower scores on the EF subscale (*β* = −0.107, 95% CI: −0.207– −0.007, *p* ≤ 0.05) and higher scores on the SE subscale (*β* = 0.110, 95% CI: 0.006–0.214, *p* ≤ 0.05; Table 3) compared to longer EBF pattern. In the adjusted model, those results remained significant (EF *β* = −0.178, 95% CI: −0.282– −0.075, *p* ≤ 0.001; SE *β* = 0.146, 95% CI: 0.037–0.255, *p* ≤ 0.01; Table 4). In addition, formula feeding pattern turned out to be associated with lower reported SE (*β* = −0.105, 95% CI: −0.208– −0.002, *p* ≤ 0.05; Table 4).

#### 3.4.2. Complementary Feeding Method

Univariate analysis revealed significant differences between scores in EF and FF subscales and method of complementary feeding introduction (Table S5). Introducing solids with the BLW method was positively associated with a score on the EF subscale (*β* = 0.112, 95% CI: 0.003–0.222, *p* ≤ 0.05), whereas using a mixed method was negatively associated with a score on the FF subscale (*β* = −0.126, 95% CI: −0.235– −0.016, *p* ≤ 0.05), when compared to the tablespoon feeding method. In multivariate analysis, only association between mixed method and score on the FF subscale remained significant (*β* = −0.124, 95% CI: −0.234– −0.013, *p* ≤ 0.05; Table 3). However, those associations were not observed in the adjusted model (Table 4).

#### 3.4.3. Types of Complementary Food Pattern

Children who were fed more often with homemade foods scored significantly higher on the SR subscale in comparison to those who ate commercial baby foods more often. This association was observed in univariate (*β* = 0.112, 95% CI: 0.021–0.202, *p* ≤ 0.05; Table S5), multivariate (*β* = 0.118, 95% CI: 0.017–0.219, *p* ≤ 0.05; Table 3) and adjusted (*β* = 0.110, 95% CI: 0.009–0.210, *p* ≤ 0.01; Table 4) models.

#### 3.4.4. Mealtime Environment Pattern

Associations between mealtime environment pattern and scores on the EF, DD, SE and FF subscales were observed in univariate (Table S5) and multivariate (Table 3) analyses. Meal consumption during distraction was negatively associated with EF (univariate: *β* = −0.240, 95% CI: −0.368– −0.112, *p* ≤ 0.001; multivariate: *β* = −0.212, 95% CI: −0.343– −0.081, *p* ≤ 0.01) and positively associated with DD (univariate: *β* = 0.185, 95% CI: 0.053–0.317, *p* ≤ 0.01; multivariate: *β* = 0.151, 95% CI: 0.018–0.285, *p* ≤ 0.05), SE (univariate: *β* = 0.178, 95% CI: 0.045–0.310, *p* ≤ 0.01; multivariate: *β* = 0.171, 95% CI: 0.035–0.307, *p* ≤ 0.05) and FF (univariate: *β* = 0.185, 95% CI: 0.054–0.316, *p* ≤ 0.01; multivariate: *β* = 0.149, 95% CI: 0.015–0.284, *p* ≤ 0.01) subscales when compared to eating meals with family. Most of these results remained significant in the adjusted model, with the exception of FF subscale (Table 4). Moreover, meal consumption separately to family was associated negatively with scores on the DD subscale (univariate: *β* = −0.191, 95% CI: −0.323– −0.059, *p* ≤ 0.01; multivariate: *β* = −0.226, 95% CI: −0.357– −0.094, *p* ≤ 0.001; adjusted: *β* = −0.224, 95% CI: −0.356– −0.092, *p* ≤ 0.001).

## 4. Discussion

This paper contributes to a growing number of studies about possible associations between nutrition in the first year and later eating behaviors. In this study, we found that early feeding factors, such as breastfeeding duration, age and method of complementary feeding introduction as well as types of complementary foods and mealtime environment may be related with eating behaviors, such as food responsiveness, enjoyment of food, desire to drink, satiety responsiveness, slowness in eating and food fussiness in children aged 1–3 years old. Results regarding subscales such as emotional over- and under-eating were not significant in all conducted analyses. We also noticed that from among sociodemographic and birth-related factors, only maternal education level, child’s age and birthweight varied in results of the CEBQ questionnaire.

### 4.1. Early Feeding Pattern—Breastfeeding and Age at Complementary Feeding Introduction

In adjusted linear regression analysis, we found that children with a longer ABF pattern scored lower on the enjoyment in food subscale when compared to those with a longer EBF pattern. This suggests that when considering this subscale, longer exclusive breastfeeding is more important than longer duration of any breastfeeding. Similarly like on the EF subscale, children with a longer ABF pattern scored lower on the desire to drink subscale when compared to longer EBF pattern children. In addition, those with a formula pattern scored higher on the desire to drink subscale. We also found that children with a longer ABF pattern scored higher on the satiety responsiveness and slowness in eating subscales, when compared to the reference group. Moreover, children with a formula pattern scored lower on the slowness in eating subscale in comparison to the longer EBF group. These results, except for enjoyment of food, are consistent with other authors’ findings, suggesting that longer breastfeeding duration may be related to lower desire to drink and higher satiety responsiveness and slowness in eating [8,20,21,22,23,53,54,55]. Admittedly, Mallan et al. [54] came to a contrary conclusion regarding satiety responsiveness, as in their study, formula-fed infants scored higher on this subscale than breastfed infants. Other authors also observed differences between breastfeeding and other subscales, such as FR (which we also found between mean scores on this subscale) and FF (inverse associations [8,21,24,25,26,53]) and EUE (positive association [8]). Nonetheless, some studies did not observe any differences [27,28].

Because with longer ABF and longer EBF patterns, age at complementary feeding introduction was very similar, the only difference between age at complementary feeding introduction was observed for the desire to drink and slowness in eating subscales (formula pattern). In the Albuquerque et al. study [56], slowness in eating was related to appetite restraint behavior, whereas desire to drink related to appetite disinhibition. However, in this study, any differences between breastfeeding duration or age at complementary feeding introduction were observed in the multivariate model. Nevertheless, in a recent study conducted by Vandyousefi et al. [55], authors observed that infants with higher slowness in eating scores had lower odds of early introduction to solids. In our study, children with a formula pattern scored lower on the slowness in eating subscale and they also had lower age at complementary feeding introduction (4.3 months), when compared to children with a longer EBF pattern, with a mean age of complementary feeding introduction of 5.6 months. Previously, other authors reported possible links between timing of complementary feeding introduction and food fussiness [21], food and satiety responsiveness [27] and enjoyment of food [27]; however, some studies observed no differences between these factors [26,56].

### 4.2. Method of Complementary Feeding Introduction

We found that children who followed a baby-led weaning method, as well as mixed method, scored higher on the enjoyment of food subscale, when compared to those children who had been introduced to solids with tablespoon method. Similarly to our results, Komninou et al. [57] reported that infants who were fed BLW, in comparison with those who were parent-fed, had higher levels of food enjoyment. Additionally, in Taylor et al. [30], authors found that mothers of infants who were introduced to solids with the BLISS method reported positive attitude on this subscale. In addition, according to mothers who used BLW or mixed method, their children scored lower on the food fussiness subscale, when compared to those who introduced solids with the tablespoon method. These findings are consistent with results from other studies [29,30,57].

Furthermore, other authors observed that BLW might be associated with lower scores on the food responsiveness subscale [21], however, results regarding satiety responsiveness are inconclusive [21,30].

### 4.3. Types of Complementary Foods

We found that infants who ate homemade complementary foods more often during the first 3 months of eating solids had a higher satiety response than those who ate commercial baby foods more often. Unfortunately, to the best of our knowledge, the number of studies regarding associations between types of complementary foods and eating behaviors in children is scarce. In the Albuquerque et al. study [56], satiety responsiveness was related to appetite restraint behaviors and authors noticed that children who ate cereals, porridge or fruit as their first solid reported more appetite restraint behaviors at 7 years old than children who received soup as their first food. Nevertheless, these data should not be directly compared to our findings as there are differences in methods between our studies.

### 4.4. Mealtime Environment

Another finding from our study is that children who followed a distracted mealtime environment pattern had lower scores on the enjoyment of food subscale when compared to those who followed the family pattern (reference group). Furthermore, they also scored higher on the desire to drink and slowness in eating subscales, when compared to the reference group. In addition, toddlers with the separated mealtime environment pattern scored lower in desire to drink subscale, when compared to the reference group. These findings were observed in the adjusted regression analysis model. In addition, when comparing mean scores on the subscales between patterns, differences were also observed on the food fussiness subscale—children with a distracted pattern scored higher in comparison to those with a family pattern. 

The same direction of observed eating behaviors was reported by Finnane et al. [18]. In their study, parents who used family meal setting practice reported higher enjoyment of food and lower food fussiness in their children. Additionally, other studies also revealed that family meals were associated with lower food fussiness [25,58]. In the Finnane et al. study [18], authors used different tools in their assessment of mealtime habits, so our results should not be directly compared; however, they also observed that parents who used persuasive feeding practices reported higher slowness in eating, food fussiness, desire to drink and lower enjoyment of food, which is in line with our observations with the distracted pattern. Similar findings were observed in other studies, where maternal pressure to eat practices were positively associated with child pickiness [32], food fussiness [31,59,60,61], desire to drink [59], slowness in eating [60,61] and negatively related to enjoyment of food [59,60,61]. Overall, this may suggest that types of mealtime environment when baby is distracted or pressured may be related to lower enjoyment of food and higher desire to drink, slowness in eating and food fussiness, as opposed to the family mealtime environment.

Inconsistency between ours and other authors’ results could be due to the different methods applied in the studies. Mallan et al. [54] used another tool in the assessment of children’s behaviors (Baby Eating Behavior Questionnaire). Additionally, in studies regarding mealtime environment, authors often used the Child Feeding Questionnaire to measure maternal practices [31,32,59,61]; nonetheless, despite this difference, the direction of observed associations is in line with ours. In addition, children’s age at assessment could also affect differences in obtained results (from 17 ± 3 weeks [54] up to 9 years [28]) and in a study conducted by Vandyousefi et al. [62], authors found low to moderate stability for selected appetite traits measured over time.

### 4.5. Possible Mechanisms

Potential mechanisms underlying possible associations between analyzed factors and eating behaviors may arise from the fact that breastfeeding, through numerous pathways, may be related with eating behaviors. Those mechanisms include maternal milk composition [63] and flavor learning [64], as well as the infant-led nature of breastfeeding, which may promote eating self-regulation [20,65]. Moreover, breastfeeding mothers may be more vigilant for satiety and hunger signals communicated by their infants [23,65,66].

Possible explanation of observed differences between complementary feeding method and eating behaviors is that parents who use BLW/mixed method usually start complementary feeding later than those who use traditional method [14,67] and Schwartz et al. [68] observed that between 5 and 7 months infants’ reactions to new foods were mostly positive. In addition, in BLW/mixed method, infants are usually introduced earlier to complex texture of solids, which can be a protective factor against food fussiness as later introduction to lumpy foods was related to feeding difficulties [69]. Interestingly, in a study conducted by Brown and Lee [21], authors noticed that the relationship between BLW and food fussiness diminished after accounting for maternal control, which suggests that a wider acceptance of foods in BLW infants may be explained by a lower level of maternal control.

The possible influence of types of complementary foods could be partially explained by differences in the nutritional quality of homemade and commercial products. According to WHO reports [15,16], commercial baby foods may have an inadequate nutritional quality, such as high content of sugars and low energy density. On the other hand, homemade meals may be more balanced and, thus, improve satiety responsiveness. Additionally, as we observed, children who were breastfed longer scored higher in satiety responsiveness. Thus, association with homemade foods can be also explained by Foterek et al. [70] findings, as they observed that children who consumed commercial foods more often showed shorter breastfeeding duration. 

As noticed by Webber et al. [61], pleasant mealtime environment (with lower maternal pressure to eat) may contribute to a child’s enjoyment in eating, which may partially explain our observations. Higher observed scores on the food fussiness subscale in children with a distracted pattern may be supported by the findings of Finnane et al. [18] who noticed that enjoyment of food and food fussiness were inversely related. Additionally, children who are distracted during mealtime may not have a chance to create desirable eating habits, as opposed to children who eat meals with other family members [7].

### 4.6. Strengths and Limitations

One of the strengths of the present study is the fact that we used a validated tool to assess children’s eating behaviors. Moreover, we took a few factors into account that gave us a broader image of possible associations between early feeding factors and children’s eating behaviors. The internet-based nature of the study ensures equal access to participate in the study for respondents from diverse regions and backgrounds. This study is also strengthened by its large sample size.

Nonetheless, our work clearly has some limitations. First, as the study was conducted among internet users, there is a possibility that more mothers who were interested in children’s nutrition were involved, so self-selection bias could have occurred (especially as we observed a relatively high percentage of exclusive breastfeeding for 6 months and complementary feeding after 4 months). Second, we asked mothers about factors related to early childhood, so memory bias could occur. Nevertheless, the recall time in our study was less than 3 years, which minimized the risk of recall bias [71,72]. Third, the study sample was characterized by an educational level that was higher than the national average; however, this could be due to the observed steady increase in the number of mothers with higher education (40% in 2010 and 50% in 2017 [73]). Moreover, in our future studies, we plan to focus on possible links between BMI, dietary habits and eating behaviors, as it was not the purpose of the present study, so we could not discuss obtained results in a broader context. In addition, not every observed difference between mean scores in given subscales was confirmed in the regression analysis, thus further studies are needed. Finally, admittedly, the original structure of the CEBQ was confirmed in a study that involved older children [34]; however, this questionnaire has also previously been used among younger children [20,30,39,74,75].

## 5. Conclusions

Taken together, our findings suggest that early feeding factors such as breastfeeding duration, types of complementary foods as well as mealtime environment in the first months of complementary feeding may be related to eating behaviors among children aged 1–3 years old, such as enjoyment of food, desire to drink, satiety responsiveness and slowness in eating.

Our findings should be considered in the context of possible implications. Eating behaviors in infancy and childhood may be an important factor in creating desirable eating habits that can be further transmitted into adulthood. Taking into account our results, parents of toddlers should receive information about the importance of breastfeeding and responsive feeding, as well as advice on how to create a mealtime environment which fosters shaping healthy eating habits. This seems to be very important, especially as some works suggest possible links between CEBQ subscales and BMI/weight status [38,59,76,77] or diet variety [78].

## Figures and Tables

**Table 1:** Sociodemographic characteristics of the study sample according to CEBQ results.

Variable	*n*	%	CEBQ Subscales							
			FR	EOE	EF	DD	SR	SE	EUE	FF
Total	467	100.0	2.05 ± 0.73	1.44 ± 0.51	3.54 ± 0.75	2.74 ± 0.87	2.92 ± 0.64	2.76 ± 0.61	2.59 ± 0.98	2.61 ± 0.90
**Maternal age (years):**										
<29	145	31.0	2.13 ± 0.84	1.47 ± 0.52	3.62 ± 0.77	2.80 ± 0.93	2.84 ± 0.68	2.77 ± 0.65	2.64 ± 0.96	2.49 ± 0.89
30–34	222	47.5	2.05 ± 0.69	1.46 ± 0.51	3.53 ± 0.74	2.77 ± 0.82	2.95 ± 0.62	2.75 ± 0.58	2.64 ± 0.97	2.66 ± 0.89
≥35	100	21.4	1.90 ± 0.63	1.37 ± 0.46	3.44 ± 0.72	2.60 ± 0.86	2.94 ± 0.64	2.81 ± 0.61	2.41 ± 1.01	2.67 ± 0.91
***p*-value**			0.147	0.213	0.112	0.169	0.478	0.747	0.077	0.221
**Maternal education:**										
high school and lower	69	14.8	2.10 ± 0.82	1.45 ± 0.49	3.61 ± 0.69	2.83 ± 0.90	2.86 ± 0.66	2.85 ± 0.70	2.59 ± 0.94	2.37 ± 0.83
university	398	85.2	2.04 ± 0.72	1.44 ± 0.51	3.53 ± 0.76	2.73 ± 0.86	2.93 ± 0.64	2.75 ± 0.59	2.59 ± 0.99	2.65 ± 0.90
***p*-value**			0.724	0.876	0.496	0.475	0.615	0.286	0.897	0.026
**Macroeconomic region residence (% GDP EU-27 average):**										
51–100	360	77.1	2.07 ± 0.73	1.43 ± 0.50	3.53 ± 0.74	2.76 ± 0.88	2.90 ± 0.65	2.77 ± 0.62	2.55 ± 0.97	2.61 ± 0.88
101–130	107	22.9	1.97 ± 0.72	1.47 ± 0.53	3.56 ± 0.76	2.67 ± 0.82	2.98 ± 0.59	2.75 ± 0.56	2.74 ± 1.02	2.60 ± 0.96
***p*-value**			0.213	0.465	0.657	0.331	0.152	0.781	0.097	0.786
**Child’s gender:**										
female	233	49.9	2.07 ± 0.71	1.47 ± 0.54	3.51 ± 0.74	2.71 ± 0.87	2.97 ± 0.61	2.80 ± 0.63	2.64 ± 0.92	2.58 ± 0.88
male	234	50.1	2.03 ± 0.76	1.42 ± 0.47	3.56 ± 0.75	2.77 ± 0.87	2.86 ± 0.67	2.73 ± 0.59	2.54 ± 1.04	2.64 ± 0.91
***p*-value**			0.345	0.693	0.622	0.353	0.114	0.294	0.177	0.482
**Child’s age (months):**										
12–18	176	37.7	2.08 ± 0.75	1.44 ± 0.49	3.73 ± 0.69 ^a^	2.68 ± 0.88	2.82 ± 0.61 ^a^	2.69 ± 0.59 ^a^	2.55 ± 1.01	2.26 ± 0.81 ^a^
19–24	120	25.7	2.03 ± 0.81	1.41 ± 0.50	3.43 ± 0.80 ^b^	2.75 ± 0.91	3.03 ± 0.67 ^b^	2.86 ± 0.64 ^b^	2.58 ± 1.02	2.78 ± 0.90 ^b^
25–36	171	36.6	2.03 ± 0.65	1.47 ± 0.52	3.42 ± 0.73 ^b^	2.81 ± 0.82	2.94 ± 0.64 ^ab^	2.77 ± 0.60 ^ab^	2.64 ± 0.93	2.84 ± 0.87 ^b^
***p*-value**			0.636	0.574	≤0.001	0.288	0.024	0.045	0.629	≤0.001

^a,b^—values with different superscript letters are significantly different (*p* ≤ 0.05); FR—food responsiveness, EOE—emotional overeating, EF—enjoyment of food, DD—desire to drink, SR—satiety responsiveness, SE—slowness in eating, EUE—emotional undereating, FF—food fussiness; GDP—gross domestic product.

**Table 2:** Early feeding practices according to CEBQ results.

Variable	*n*	%	CEBQ Subscales							
			FR	EOE	EF	DD	SR	SE	EUE	FF
**Early feeding pattern:**										
longer ABF	174	37.1	1.91 ± 0.65 ^a^	1.43 ± 0.46	3.50 ± 0.69	2.55 ± 0.80 ^a^	3.00 ± 0.56 ^a^	2.83 ± 0.60	2.63 ± 1.01	2.54 ± 0.85
formula	160	34.3	2.09 ± 0.76 ^ab^	1.41 ± 0.50	3.50 ± 0.85	2.89 ± 0.93 ^b^	2.89 ± 0.73 ^ab^	2.70 ± 0.63	2.56 ± 0.99	2.63 ± 0.96
longer EBF	133	28.5	2.17 ± 0.78 ^b^	1.50 ± 0.56	3.63 ± 0.69	2.83 ± 0.83 ^b^	2.83 ± 0.63 ^b^	2.75 ± 0.60	2.58 ± 0.94	2.67 ± 0.87
***p*-value**			0.009	0.434	0.298	0.002	0.009	0.173	0.805	0.489
**Age at CFI (months):**										
<4	54	11.6	2.03 ± 0.77	1.37 ± 0.49	3.29 ± 0.84	3.03 ± 1.14	3.05 ± 0.80	2.77 ± 0.70 ^ab^	2.69 ± 1.02	2.89 ± 0.98
4–6	380	81.4	2.05 ± 0.74	1.45 ± 0.52	3.58 ± 0.74	2.71 ± 0.83	2.90 ± 0.62	2.74 ± 0.59 ^a^	2.58 ± 0.98	2.56 ± 0.88
≥ 7	33	7.1	2.00 ± 0.63	1.45 ± 0.39	3.52 ± 0.62	2.66 ± 0.69	2.93 ± 0.57	3.03 ± 0.61 ^b^	2.54 ± 0.91	2.69 ± 0.87
***p*-value**			0.933	0.260	0.062	0.283	0.611	0.030	0.821	0.051
**CF method:**										
BLW	134	28.7	2.03 ± 0.71	1.41 ± 0.45	3.66 ± 0.76 ^a^	2.62 ± 0.78	2.98 ± 0.56	2.76 ± 0.60	2.51 ± 0.96	2.50 ± 0.90 ^a^
mixed	141	30.2	2.06 ± 0.79	1.49 ± 0.54	3.63 ± 0.72 ^a^	2.74 ± 0.86	2.86 ± 0.61	2.70 ± 0.58	2.59 ± 0.94	2.45 ± 0.81 ^a^
TSF	192	41.1	2.04 ± 0.71	1.43 ± 0.52	3.38 ± 0.73 ^b^	2.84 ± 0.92	2.92 ± 0.71	2.81 ± 0.64	2.64 ± 1.02	2.80 ± 0.92 ^b^
***p*-value**			0.984	0.501	≤0.001	0.111	0.310	0.560	0.446	≤0.001
**Types of complementary food pattern:**										
homemade	257	55.0	2.05 ± 0.73	1.48 ± 0.52	3.59 ± 0.74	2.69 ± 0.81	2.98 ± 0.59	2.79 ± 0.59	2.55 ± 0.96	2.56 ± 0.88
commercial	210	45.0	2.04 ± 0.73	1.40 ± 0.49	3.48 ± 0.75	2.81 ± 0.93	2.84 ± 0.69	2.74 ± 0.63	2.64 ± 1.00	2.66 ± 0.92
***p*-value**			0.923	0.079	0.113	0.408	0.011	0.477	0.434	0.203
**Mealtime environment pattern:**										
distracted	55	11.8	2.07 ± 0.67	1.51 ± 0.55	3.13 ± 0.71 ^a^	3.02 ± 1.06 ^a^	3.05 ± 0.65	2.97 ± 0.60 ^a^	2.83 ± 1.07	2.97 ± 0.85 ^a^
separated	115	24.6	2.05 ± 0.75	1.42 ± 0.47	3.33 ± 0.78 ^a^	2.59 ± 0.83 ^b^	2.94 ± 0.71	2.73 ± 0.64 ^b^	2.51 ± 0.98	2.74 ± 0.99 ^ab^
family	297	63.6	2.04 ± 0.74	1.44 ± 0.51	3.69 ± 0.70 ^b^	2.75 ± 0.83 ^ab^	2.88 ± 0.61	2.74 ± 0.59 ^b^	2.58 ± 0.96	2.49 ± 0.84 ^b^
***p*-value**			0.816	0.568	≤0.001	0.035	0.157	0.026	0.189	≤0.001

^a,b^—values with different superscript letters are significantly different (*p* ≤ 0.05); FR—food responsiveness, EOE—emotional overeating, EF—enjoyment of food, DD—desire to drink, SR—satiety responsiveness, SE—slowness in eating, EUE—emotional undereating, FF—food fussiness; ABF—any breastfeeding; EBF—exclusive breastfeeding; Early feeding patterns: longer ABF—currently breastfed, EBF duration ~4.1 months, age at CFI ~5.6 months; formula—not currently breastfed, EBF duration ~0.3 months, age at CFI ~4.3 months; longer EBF—not currently breastfed, EBF duration ~5.4 months, age at CFI ~5.6 months; CFI—complementary feeding introduction; CF—complementary feeding; BLW—baby led weaning; TSF—tablespoon feeding.

**Table 3:** Multivariate regression analysis predicting eating behaviors.

Factors	CEBQ Subscales							
	FR *β* (95% CI)	EOE *β* (95% CI)	EF *β* (95% CI)	DD *β* (95% CI)	SR *β* (95% CI)	SE *β* (95% CI)	EUE *β* (95% CI)	FF *β* (95% CI)
**Early feeding pattern:**								
longer ABF	−0.169 (−0.273–0.064)	−0.035 (−0.139–0.069)	−0.107 (−0.207–−0.007) *	−0.187 (−0.289–−0.085) ***	0.117 (0.014–0.220) *	0.110 (0.006–0.214) *	0.045 (−0.060–0.149)	−0.024 (−0.126–0.079)
formula	0.044 (−0.059–0.147)	−0.043 (−0.146–0.060)	0.005 (−0.094–0.104)	0.114 (0.013–0.215) **	−0.018 (−0.120–0.084)	−0.084 (−0.187–0.019)	−0.036 (−0.140–0.068)	−0.016 (−0.118–0.085)
longer EBF	Ref	Ref	Ref	Ref	Ref	Ref	Ref	Ref
**CF method:**								
BLW	0.012 (−0.117–0.142)	−0.097 (−0.227–0.032)	0.026 (−0.098–0.151)	−0.090 (−0.218–0.037)	0.044 (−0.085–0.173)	−0.025 (−0.155–0.104)	−0.051 (−0.181–0.079)	−0.013 (−0.141–0.114)
mixed	0.018 (−0.094–0.131)	0.095 (−0.017–0.208)	0.083 (−0.025–0.190)	0.021 (−0.089–0.132)	−0.057 (−0.168–0.055)	−0.059 (−0.171–0.052)	0.014 (−0.099–0.126)	−0.124 (−0.234–−0.013) *
TSF	Ref	Ref	Ref	Ref	Ref	Ref	Ref	Ref
**Types of complementary food pattern:**								
homemade	0.021 (−0.081–0.123)	0.108 (0.000–0.210)	−0.003 (−0.101–0.095)	−0.014 (−0.115–0.086)	0.118 (0.017–0.219)*	0.059 (−0.043–0.160)	−0.030 (−0.133–0.073)	0.002 (−0.099–0.103)
commercial	Ref	Ref	Ref	Ref	Ref	Ref	Ref	Ref
**Mealtime environment pattern:**								
distracted	0.022 (−0.115–0.158)	0.092 (−0.044–0.229)	−0.212 (−0.343–−0.081) **	0.151 (0.018–0.285) *	0.122 (−0.013–0.257)	0.171 (0.035–0.307) *	0.125 (−0.012–0.262)	0.149 (0.015–0.284) **
separated	−0.015 (−0.149–0.119)	−0.071 (−0.205–0.064)	−0.068 (−0.197–0.061)	−0.226 (−0.357–−0.094) ***	0.010 (−0.123–0.143)	−0.101 (−0.234–0.033)	−0.121 (−0.255–0.014)	0.004 (−0.128–0.136)
family	Ref	Ref	Ref	Ref	Ref	Ref	Ref	Ref
**R^2^**	0.01	0.01	0.09 ***	0.05 ***	0.03 **	0.02 *	0.00	0.04 ***

FR—food responsiveness, EOE—emotional overeating, EF—enjoyment of food, DD—desire to drink, SR—satiety responsiveness, SE—slowness in eating, EUE—emotional undereating, FF—food fussiness; CF—complementary feeding; Early feeding patterns: longer ABF—currently breastfed, EBF duration ~4.1 months, age at CFI ~5.6 months; formula—not currently breastfed, EBF duration ~0.3 months, age at CFI ~4.3 months; longer EBF—not currently breastfed, EBF duration ~5.4 months, age at CFI ~5.6 months; BLW—baby-led weaning; TSF—tablespoon feeding; * *p* ≤ 0.05; ** *p* ≤ 0.01; *** *p* ≤ 0.001.

**Table 4:** Adjusted regression analysis predicting eating behaviors.

Factors	CEBQ Subscales							
	FR *β* (95% CI)	EOE *β* (95% CI)	EF *β* (95% CI)	DD *β* (95% CI)	SR *β* (95% CI)	SE *β* (95% CI)	EUE *β* (95% CI)	FF *β* (95% CI)
**Early feeding pattern:**								
longer ABF	−0.197 (−0.307–0.087)	−0.029 (−0.139–0.082)	−0.178 (−0.282–−0.075) ***	−0.183 (−0.292–−0.074) **	0.158 (0.050–0.267) **	0.146 (0.037–0.255) **	0.064 (–0.047–0.175)	0.062 (−0.042–0.166)
formula	0.047 (−0.058–0.151)	−0.042 (–0.146–0.063)	0.018 (−0.080–0.116)	0.109 (0.006–0.212) *	−0.026 (−0.128–0.076)	−0.105 (−0.208–−0.002) *	−0.039 (−0.144–0.067)	−0.025 (−0.124–0.073)
longer EBF	Ref	Ref	Ref	Ref	Ref	Ref	Ref	Ref
**CF method:**								
BLW	0.013 (−0.117–0.143)	−0.100 (−0.231–0.030)	0.035 (−0.087–0.157)	−0.090 (−0.218–0.038)	0.036 (−0.092–0.163)	−0.036 (−0.164–0.093)	−0.056 (−0.187–0.075)	−0.017 (−0.139–0.105)
mixed	0.010 (−0.102–0.123)	0.095 (−0.018–0.208)	0.064 (−0.042–0.169)	0.022 (−0.089–0.133)	−0.044 (−0.155–0.066)	−0.052 (−0.163–0.059)	0.016 (−0.097–0.130)	−0.096 (−0.202–0.010)
TSF	Ref	Ref	Ref	Ref	Ref	Ref	Ref	Ref
**Types of complementary food pattern:**								
homemade	0.025 (−0.077–0.127)	0.108 (0.000–0.210)	0.008 (−0.088–0.104)	−0.014 (−0.115–0.086)	0.110 (0.009–0.210) **	0.053 (−0.048–0.154)	−0.032 (−0.135–0.071)	−0.013 (−0.109–0.083)
commercial	Ref	Ref	Ref	Ref	Ref	Ref	Ref	Ref
**Mealtime environment pattern:**								
distracted	0.030 (−0.107–0.166)	0.092 (−0.046–0.229)	−0.192 (−0.321–−0.064) **	0.150 (0.015–0.285) *	0.110 (−0.024–0.244)	0.160 (0.025–0.295) *	0.120 (−0.017–0.258)	0.125 (−0.004–0.253)
separated	−0.019 (−0.153–0.115)	−0.071 (−0.206–0.064)	−0.078 (−0.205–0.048)	−0.224 (−0.356– −0.092) ***	0.016 (−0.116–0.147)	−0.093 (−0.026–0.039)	−0.119 (−0.254–0.016)	0.018 (−0.109–0.144)
family	Ref	Ref	Ref	Ref	Ref	Ref	Ref	Ref
**R^2^**	0.01	0.00	0.12 ***	0.04 **	0.05 ***	0.03 **	0.00	0.12 ***

FR—food responsiveness, EOE—emotional overeating, EF—enjoyment of food, DD—desire to drink, SR—satiety responsiveness, SE—slowness in eating, EUE—emotional undereating, FF—food fussiness; CF—complementary feeding; Early feeding patterns: longer ABF—currently breastfed, EBF duration ~4.1 months, age at CFI ~5.6 months; formula—not currently breastfed, EBF duration ~0.3 months, age at CFI ~4.3 months; longer EBF—not currently breastfed, EBF duration ~5.4 months, age at CFI ~5.6 months; BLW—baby-led weaning; TSF—tablespoon feeding; * *p* ≤ 0.05; ** *p* ≤ 0.01; *** *p* ≤ 0.001; model adjusted for children age, gender and maternal education.

## Data Availability

The data presented in this study are available on request from the corresponding author.

## Supplementary Materials

The following supporting information can be downloaded at: https://www.mdpi.com/article/10.3390/nu14112279/s1; Table S1: Component variables in k-means analysis—early feeding pattern; Table S2: Component variables in k-means analysis—types of complementary food pattern; Table S3: Component variables in k-means analysis—mealtime environment pattern; Table S4: Birth-related factors, maternal BMI and ever formula feeding according to CEBQ results; Table S5: Univariate regression analysis predicting eating behaviors.
