# Supplementary material for: Early Feeding Factors and Eating Behaviors among Children Aged 1–3: A Cross-Sectional Study
Source: Nutrients. 2022 May 29;14(11):2279. doi: 10.3390/nu14112279 (PMC9183102; doi:10.3390/nu14112279)
Supplement: Supplementary file 1 [file nutrients-14-02279-s001.zip › nutrients-1742435-supplementary.pdf]

nutrients

MDPI

Article

Early Feeding Factors and Eating Behaviors Among Children Aged 1-3: A Cross-Sectional Study

Daria Masztalerz-Kozubek, Monika A. Zielinska-Pukos* and Jadwiga Hamulka

Table S1. Component variables in k-means analysis – early feeding pattern.

Variable	Early feeding pattern (%)			p-value
	Longer ABF (n=174)	Formula (n=160)	Longer EBF (n=133)	
**Current breastfeeding:**	yes 100%	no 100%	no 100%	≤0.001
**EBF duration (months):**				
never BF/EBF <1	23.0	85.0	0.0	
1-4	13.2	15.0	21.0	≤0.001
5-7	63.8	0.0	79.0	
**Age at CFI (months):**				
<4	2.9	29.4	1.5	
4-6	86.8	69.4	88.7	≤0.001
≥ 7	10.3	1.2	9.8	

ABF – any breastfeeding; BF – breastfeeding; EBF – exclusive breastfeeding; CFI – complementary feeding introduction

Table S2. Component variables in k-means analysis – types of complementary food pattern.

Frequency of consumption of selected foods during the first 3 months of complementary feeding	Types of complementary food pattern (%)		p-value
	**Homemade** (n=257)	**Commercial** (n=210)	
**Commercial soup/dinner jars:**			
never or almost never	56.9	1.9	
once a month or less	20.2	2.9	
few times per month	20.2	21.4	
few times per week	2.3	45.7	≤0.001
everyday	0.4	25.2	
few times per day	0.0	2.9	
I don't remember	0.0	0.0	
**Commercial baby cereals:**			
never or almost never	67.7	11.4	
once a month or less	15.2	6.2	
few times per month	10.5	18.1	
few times per week	4.7	40.0	≤0.001
everyday	1.9	22.4	
few times per day	0.0	1.9	
I don't remember	0.0	0.0	
**Commercial fruit jars:**			
never or almost never	40.8	2.4	
once a month or less	21.4	1.9	
few times per month	25.7	22.9	
few times per week	11.7	49.0	≤0.001
everyday	0.4	20.9	
few times per day	0.0	2.4	
I don't remember	0.0	0.5	
**Commercial baby juices/bevereges:**			
never or almost never	0.4	7.6	
once a month or less	0.8	5.2	
few times per month	0.4	1.9	
few times per week	0.0	1.0	≤0.001
everyday	0.0	0.5	
few times per day	95.3	79.0	
I don't remember	3.1	4.8	
**Commercial tea for babies:**			
never or almost never	98.8	89.0	
once a month or less	0.4	1.9	
few times per month	0.8	4.8	
few times per week	0.0	2.9	≤0.001
everyday	0.0	0.9	

few times per day	0.0	0.5	
I don't remember	0.0	0.0	

Table S2. Component variables in k-means analysis – types of complementary food pattern – *cont.*

**Frequency of consumption of selected foods during the first 3 months of complementary feeding**	**Types of complementary food pattern (%)**		**p-value**
	**Homemade** (n=257)	**Commercial** (n=210)	
**Homemade meals cooked especially for baby:**			
never or almost never	15.9	6.7	
once a month or less	9.3	3.8	
few times per month	19.5	13.8	
few times per week	17.9	37.6	≤0.001
everyday	23.4	29.0	
few times per day	13.6	8.1	
I don't remember	0.4	1.0	
**Family meals adjusted for baby:**			
never or almost never	2.7	11.4	
once a month or less	1.6	6.2	
few times per month	5.8	19.1	
few times per week	15.6	32.9	≤0.001
everyday	37.0	24.3	
few times per day	34.2	5.2	
I don't remember	3.1	0.9	
**Family meals not adjusted for baby:**			
never or almost never	77.4	73.8	
once a month or less	10.9	13.8	
few times per month	6.2	5.7	
few times per week	2.7	3.3	0.930
everyday	1.2	1.0	
few times per day	0.4	1.0	
I don't remember	1.2	1.4	

Table S3. Component variables in k-means analysis – mealtime environment pattern.

Frequency of meals consumption in a given way	Mealtime environment pattern (%)			p-value
	**Distracted** (n=55)	**Separate** (n=115)	**Family** (n=297)	
**In the same time than other family members:**				
never or almost never	3.6	22.6	0.0	
occasionally	34.6	70.4	0.3	
often	38.2	5.2	39.1	≤0.001
always/almost always	21.8	0.0	60.6	
I don't remember	1.8	1.8	0.0	
**Separately from other family members:**				
never or almost never	10.9	1.7	18.9	
occasionally	34.6	4.4	66.0	
often	52.7	60.9	13.8	≤0.001
always/almost always	1.8	33.0	0.3	
I don't remember	0.0	0.0	1.0	
**During watching TV:**				
never or almost never	52.7	87.0	94.3	
occasionally	30.9	11.3	4.7	
often	12.7	1.7	1.0	≤0.001
always/almost always	3.7	0.0	0.0	
I don't remember	0.0	0.0	0.0	
**When distracted:**				
never or almost never	0.0	91.3	97.6	
occasionally	70.9	8.7	2.4	
often	29.1	0.0	0.0	≤0.001
always/almost always	0.0	0.0	0.0	
I don't remember	0.0	0.0	0.0	
**During playtime:**				
never or almost never	36.4	91.3	89.2	
occasionally	40.0	4.3	9.4	
often	23.6	2.6	1.4	≤0.001
always/almost always	0.0	0.9	0.0	
I don't remember	0.0	0.9	0.0	
**During sleeping:**				
never or almost never	89.1	95.7	99.0	
occasionally	7.3	2.6	0.7	
often	3.6	1.7	0.3	0.002
always/almost always	0.0	0.0	0.0	

I don't remember	0.0	0.0	0.0	
**Until child's finished meal:**				
never or almost never	60.0	88.7	94.0	
occasionally	25.5	7.8	4.7	
often	9.1	3.5	0.3	≤0.001
always/almost always	3.6	0.0	0.3	
I don't remember	1.8	0.0	0.7	

Table S4. Birth-related factors, maternal BMI and ever formula feeding according to CEBQ results.

Variable	n	%	CEBQ subscales							
			FR	EOE	EF	DD	SR	SE	EUE	FF
**Pregnancy duration:**										
preterm	42	9.0	1.88 ± 0.63	1.49 ± 0.59	3.39 ± 0.79	2.62 ± 0.72	3.11 ± 0.83	2.64 ± 0.62	2.75 ± 1.10	2.73 ± 0.87
term	425	91.0	2.06 ± 0.74	1.44 ± 0.50	3.55 ± 0.74	2.76 ± 0.88	2.90 ± 0.62	2.78 ± 0.61	2.57 ± 0.97	2.60 ± 0.90
**p-value**			0.123	0.856	0.276	0.440	0.114	0.135	0.364	0.446
**Birthweight to gestational age:**										
SGA	17	3.6	1.99 ± 0.58	1.54 ± 0.63	3.50 ± 0.94	2.90 ± 0.77	3.25 ± 0.62	2.93 ± 0.60	2.90 ± 1.02	2.46 ± 0.80
AGA	360	77.1	2.05 ± 0.75	1.45 ± 0.52	3.54 ± 0.76	2.71 ± 0.88	2.91 ± 0.64	2.75 ± 0.62	2.56 ± 0.99	2.63 ± 0.91
LGA	90	19.3	2.06 ± 0.69	1.39 ± 0.43	3.54 ± 0.69	2.86 ± 0.83	2.88 ± 0.66	2.79 ± 0.57	2.64 ± 0.95	2.56 ± 0.87
**p-value**			0.961	0.654	0.929	0.204	0.133	0.525	0.305	0.727
**Birthweight (g):**										
< 2500	23	4.9	1.64 ± 0.45[a]	1.39 ± 0.45	3.21 ± 0.70	2.78 ± 0.76	3.17 ± 0.73	2.62 ± 0.64	2.85 ± 1.14	2.64 ± 0.91
2500-3999	401	85.9	2.07 ± 0.75[b]	1.46 ± 0.52	3.56 ± 0.75	2.75 ± 0.88	2.91 ± 0.64	2.76 ± 0.61	2.57 ± 0.97	2.61 ± 0.90
≥ 4000	43	9.2	2.02 ± 0.65[ab]	1.36 ± 0.40	3.51 ± 0.69	2.68 ± 0.76	2.81 ± 0.57	2.90 ± 0.59	2.60 ± 0.98	2.54 ± 0.86
**p-value**			0.016	0.643	0.092	0.904	0.151	0.118	0.589	0.886
**Maternal BMI:**										
< 18.5	33	7.1	2.09 ± 0.78	1.56 ± 0.66	3.56 ± 0.65	2.71 ± 0.79	3.03 ± 0.52	2.80 ± 0.55	2.39 ± 0.95	2.63 ± 0.84
18.5-24.9	286	61.2	2.01 ± 0.73	1.42 ± 0.50	3.53 ± 0.75	2.72 ± 0.86	2.92 ± 0.65	2.77 ± 0.58	2.63 ± 0.99	2.64 ± 0.92
25-29.9	97	20.8	2.08 ± 0.76	1.47 ± 0.49	3.54 ± 0.77	2.79 ± 0.92	2.86 ± 0.64	2.75 ± 0.65	2.62 ± 1.00	2.59 ± 0.86
≥ 30	51	10.9	2.13 ± 0.64	1.43 ± 0.47	3.54 ± 0.78	2.80 ± 0.89	2.93 ± 0.65	2.74 ± 0.71	2.44 ± 0.89	2.45 ± 0.86
**p-value**			0.486	0.641	0.999	0.840	0.498	0.929	0.425	0.659
**Ever formula feeding:**										
no	269	57.6	2.05 ± 0.75	1.46 ± 0.51	3.53 ± 0.73	2.72 ± 0.88	2.95 ± 0.63	2.78 ± 0.61	2.59 ± 0.97	2.65 ± 0.90

yes	198	42.4	2.04 ± 0.72	1.42 ± 0.50	3.58 ± 0.77	2.79 ± 0.85	2.86 ± 0.66	2.74 ± 0.61	2.59 ± 1.02	2.52 ± 0.87
**p-value**			0.950	0.419	0.372	0.362	0.233	0.484	0.965	0.144

[a,b] – values with different superscript letters are significantly different (p≤0.05); FR – food responsiveness, EOE – emotional overeating, EF – enjoyment of food, DD – desire to drink, SR – satiety responsiveness, SE – slowness in eating, EUE – emotional undereating, FF – food fussiness; SGA – small for gestational age; AGA – appropriate for gesta- tional age; LGA – large for gestational age; BMI – body mass index

Table S5. Univariate regression analysis predicting eating behaviors.

Factors	CEBQ subscales							
	**FR** *β* (95% CI)	**EOE** *β* (95% CI)	**EF** *β* (95% CI)	**DD** *β* (95% CI)	**SR** *β* (95% CI)	**SE** *β* (95% CI)	**EUE** *β* (95% CI)	**FF** *β* (95% CI)
**Early feeding pattern:**								
longer ABF	-0.160 (-0.260– -0.060)**	-0.031 (-0.132–0.071)	-0.046 (-0.147–0.055)	-0.194 (-0.294– -0.094)***	0.120 (0.019–0.221)	0.097 (-0.005–0.198)	0.030 (–0.071–0.132)	-0.067 (-0.168–0.034)
formula	0.038 (-0.063–0.138)	-0.052 (-0.153–0.049)	-0.047 (-0.148–0.054)	0.119 (0.019–0.219)*	-0.022 (-0.123–0.079)	-0.074 (-0.175–0.027)	-0.022 (-0.124–0.079)	0.019 (-0.082–0.120)
longer EBF	Ref	Ref	Ref	Ref	Ref	Ref	Ref	Ref
**R^2**	0.02**	0.01	0.01	0.03***	0.01	0.01	0.00	0.00
**CF method:**								
BLW	-0.015 (-0.126–0.097)	-0.056 (-0.167–0.056)	0.112 (0.003–0.222)*	-0.108 (-0.219–0.003)	0.077 (-0.034–0.189)	0.003 (-0.108–0.114)	-0.057 (-0.168–0.054)	-0.076 (-0.186–0.034)
mixed	0.016 (-0.096–0.127)	0.074 (-0.037–0.186)	0.083 (-0.027–0.193)	0.007 (-0.104–0.118)	-0.076 (-0.187–0.035)	-0.078 (-0.189–0.033)	0.007 (-0.104–0.118)	-0.126 (-0.235– -0.016)*
TSF	Ref	Ref	Ref	Ref	Ref	Ref	Ref	Ref
**R^2**	0.00	0.00	0.03***	0.01	0.00	0.00	0.00	0.03***
**Types of complementary food pattern:**								
homemade	0.008 (-0.083–0.100)	0.076 (-0.015–0.167)	0.070 (-0.020–0.161)	-0.067 (-0.158–0.024)	0.112 (0.021–0.202)*	0.040 (-0.051–0.131)	-0.046 (-0.137–0.045)	-0.056 (-0.147–0.035)
commercial	Ref	Ref	Ref	Ref	Ref	Ref	Ref	Ref
**R^2**	0.00	0.00	0.00	0.00	0.010*	–0.00	0.00	0.00

**Mealtime environment pattern:**								
distracted	0.015 (-0.119–0.148)	0.076 (-0.057–0.209)	-0.240 (-0.368– -0.112)***	0.185 (0.053–0.317)**	0.103 (-0.030–0.236)	0.178 (0.045–0.310)**	0.137 (0.000–0.270)	0.185 (0.054–0.316)**
separated	0.000 (-0.133–0.133)	-0.059 (-0.192–0.074)	-0.057 (-0.185–0.070)	-0.191 (-0.323– -0.059)**	-0.020 (-0.153–0.112)	-0.116 (-0.248–0.016)	-0.115 (-0.248–0.017)	0.004 (-0.127–0.135)
family	Ref	Ref	Ref	Ref	Ref	Ref	Ref	Ref
**R^2**	0.00	0.00	0.08***	0.02*	0.00	0.01*	0.00	0.03***

FR – food responsiveness, EOE – emotional overeating, EF – enjoyment of food, DD – desire to drink, SR – satiety responsiveness, SE – slowness in eating, EUE – emotional under- eating, FF – food fussiness; CF – complementary feeding; Early feeding patterns: longer ABF – currently breastfed, EBF duration ~4.1 months, age at CFI ~5.6 months; formula – not currently breastfed, EBF duration ~0.3 months, age at CFI ~4.3 months; longer EBF – not currently breastfed, EBF duration ~5.4 months, age at CFI ~5.6 months; BLW – baby-led weaning; TSF – tablespoon feeding; *p ≤ 0.05; **p ≤ 0.01; ***p ≤ 0.001
